# MD Simulations on a Well-Built Docking Model Reveal Fine Mechanical Stability and Force-Dependent Dissociation of Mac-1/GPIbα Complex

**DOI:** 10.3389/fmolb.2021.638396

**Published:** 2021-04-22

**Authors:** Xiaoyan Jiang, Xiaoxi Sun, Jiangguo Lin, Yingchen Ling, Ying Fang, Jianhua Wu

**Affiliations:** ^1^ Institute of Biomechanics/School of Biology and Biological Engineering, South China University of Technology, Guangzhou, China; ^2^ Research Department of Medical Sciences, Guangdong Provincial People’s Hospital, Guangdong Academy of Medical Sciences, Guangzhou, China

**Keywords:** Mac-1, GPIbα, molecular dynamics simulation, structure–function relation, leukocyte–platelet interaction

## Abstract

Interaction of leukocyte integrin macrophage-1 antigen (Mac-1) to platelet glycoprotein Ibα (GPIbα) is critical for platelet–leukocyte crosstalk in hemostasis and inflammatory responses to vessel injuries under hemodynamic environments. The mechano-regulation and its molecular basis for binding of Mac-1 to GPIbα remain unclear, mainly coming from the lack of crystal structure of the Mac-1/GPIbα complex. We herein built a Mac-1/GPIbα complex model through a novel computer strategy, which included a flexible molecular docking and system equilibrium followed by a “force-ramp + snapback” molecular dynamics (MD) simulation. With this model, a series of “ramp-clamp” steered molecular dynamics (SMD) simulations were performed to examine the GPIbα–Mac-1 interaction under various loads. The results demonstrated that the complex was mechano-stable for both the high rupture force (>250 pN) at a pulling velocity of 3 Å/ns and the conformational conservation under various constant tensile forces (≤75 pN); a catch-slip bond transition was predicted through the dissociation probability, examined with single molecular AFM measurements, reflected by the interaction energy and the interface H-bond number, and related to the force-induced allostery of the complex; besides the mutation-identified residues D222 and R218, the residues were also dominant in the binding of Mac-1 to GPIbα. This study recommended a valid computer strategy for building a likely wild-type docking model of a complex, provided a novel insight into the mechanical regulation mechanism and its molecular basis for the interaction of Mac-1 with GPIbα, and would be helpful for understanding the platelet–leukocyte interaction in hemostasis and inflammatory responses under mechano-microenvironments.

## Introduction

Interaction of platelet glycoprotein receptor I bα (GPIbα) with α_M_β_2_ (Mac-1) integrin mediates crosstalk between platelets and leukocytes in hemostasis and inflammatory responses to the vessel damages (Diamond et al., 1995). In platelet recruitment and thrombus formation, the circulating platelets first tether to, then roll on, and lastly adhered at the injured vessel sites, companying with platelet activation (Rahman and Hlady, 2019). The activated platelets recruit and activate leukocytes to prevent infection caused by an injury (Von Hundelshausen and Weber, 2007; Karshovska et al., 2013; Schrottmaier et al., 2015). Binding of P-selectin on activated platelet to P-selectin glycoprotein ligand 1 (PSGL-1) mediates adhesion of leukocytes to the platelets and further induces activation of Mac-1 integrin on leukocytes. The activated Mac-1 integrin enhances firm adhesion of leukocytes to platelets through binding with GPIbα (Diacovo et al., 1996; McEver and Cummings, 1997). The interaction of Mac-1 to GPIbα may be force dependent, especially under pathological flow environments (Kruss et al., 2013).

Mac-1 integrin, a heterodimeric protein, includes α and β subunits, regulates leukocyte functions, including crawling (Phillipson et al., 2006; Sumagin et al., 2010), chemotaxis (McDonald et al., 2010), survival (Whitlock et al., 2000), apoptosis (Coxon et al., 1996), and neutrophil extracellular trap (NET) formation (Behnen et al., 2014; Silva et al., 2020). The major binding site locates at the inserted (I) domain of the αM subunit (Diamond et al., 1993), like the A1 domain of von Willebrand factor (VWF) (Sadler et al., 1985). GPIbα is composed of an N-terminal, a transmembrane, and an intracellular domain. The N-terminal domain exhibits a narrow and curved shape, and eight leucine-rich repeats (LRRs) constitute the central region of the molecule, belonging to the typical LRR protein (Kobe and Kajava, 2001). Binding of N-terminal region (F201–G268) of GPIbα to the I-domain of Mac-1 induces stable association of platelets with neutrophils (Simon et al., 2000; Wang et al., 2005), while deletion or inhibition of either Mac-1 or GPIbα suppresses interaction of platelets with neutrophils or monocytes under inflammatory conditions (Hidalgo et al., 2009). Mutation experiments suggest that the four residues, such as T211, T213, R216, and R244 on Mac-1, locate at the binding site of the complex, and so do the residues R218, R222, and N223 on GPIbα (Simon et al., 2000; Ehlers et al., 2003; Wang et al., 2005; Morgan et al., 2019). Mutations of the residues T213 and R216 on Mac-1 cause delay of thrombosis after carotid and cremaster muscle microvascular injury (Ehlers et al., 2003), suggesting promotion of GPIbα–Mac-1 interaction to thrombosis.

Studies *in vitro* have shown that increasing wall shear stress in the range from 0.1 to 5 dyn/cm^2^ reduces the attachments of neutrophils on GPIbα-coated substrates (Kruss et al., 2013), but less knowledge is about mechano-regulation on the interaction of Mac-1 with GPIbα. Lack of crystal structure of the complex makes the molecular basis of Mac-1/GPIbα interaction unclear, despite the main crystallized structures of Mac-1 and GPIbα have respectively been solved (Ehlers et al., 2003; Li and Emsley, 2013). Molecular docking is demonstrated to be a powerful tool in building a computer model of ligand–receptor complex for various adhesive molecular systems, while a very likely bad thermo- and mechano-stability make the docking model unreliable (Zeng et al., 2013). However, AFM measurements reveal successfully various force-dependent ligand–receptor interactions of adhesive (Lee et al., 2016; Li et al., 2018) and so do the molecular dynamics (MD) simulations (Fang et al., 2012; Fang et al., 2018).

We herein built a model of the Mac-1/GPIbα complex through a novel computer strategy, in which a flexible molecular docking follows a “force-ramp + snapback” MD simulation (Methods and Materials) (Smith et al., 2005; Torchala et al., 2013). This model was predicted to be a likely wild-type one for its thermo- and mechano-stability and used to examine the mechano-regulation mechanism and its molecular basis of interaction of Mac-1 to GPIbα by running a series of “ramp-clamp” steered molecular dynamics (SMD) simulations. A biphasic force-dependent dissociation of Mac-1 from GPIbα was predicted by MD simulations, examined through AFM measurements, and demonstrated to be relative to force-induced allostery of the complex. The present computer strategy for optimizing the docking model with the treatment of “force-ramp + snapback” SMD simulation might be served as a novel powerful tool in building a likely wild-type docking model of complex for various adhesive molecular systems. Besides, the present study provides a novel insight into the mechano-regulation mechanism and its molecular basis for the interaction of Mac-1 to GPIbα, and further is helpful to understand the effects of force on platelet–leukocyte crosstalk in hemostasis and inflammatory responses under flows.

## Materials and Methods

### AFM Bond Lifetime Measurement Experiments

Recombinant human integrin α_M_β_2_ (Mac-1) and recombinant human CD42b (GPIbα) were purchased from R&D Systems. Anti-6× His tag antibody was purchased from Abcam (Supplement Material). MnCl_2_ and BSA were purchased from Sigma-Aldrich. To measure the interaction of Mac-1–GPIbα, a cantilever tip (MLCT; Bruker AFM Probes) was incubated with 30 μl of 15 μg/ml Mac-1 overnight at 4°C. 30 μl of GPIbα (15 μg/ml) was adsorbed on a small spot on a petri dish overnight at 4°C. After rinsing with PBS, the tip of the cantilever was incubated with HBSS containing 2% BSA and 1 mM Mn^2+^ for an hour at room temperature to obtain activated Mac-1. After rinsing with PBS, the petri dish was incubated for 30 min at room temperature with HBSS containing 2% BSA to block nonspecific adhesion. Notably, Mac-1 and GPIbα were also immobilized on a cantilever tip and a petri dish surface by the anti-6× His tag antibody capturing to examine the effect of molecule orientations on interactions (Supplement Material). During each measurement cycle, a petri dish was driven by the piezoelectric translator to contact with a cantilever tip to reach the set-point (0.5 V), and then immediately retracted slightly and held close to the tip for 0.5 s to allow bond formation and retracted along the z direction at a speed of 200 nm/s. A feedback system was applied in the experiments. During the retraction, if a tensile force was detected (adhesion) and reached the preset level, the retraction would stop to clamp the force at that level until the tensile force broke and further retracted to its initial position. If no tensile force was detected (no adhesion) or a tensile force did not reach the preset level, the petri dish was directly retracted to its initial position. The number of adhesion events and bond lifetimes at desired forces were measured from the force–time curves.

### Molecular Docking

Flexible docking of I-domain of Mac-1 (residues 131–A317; PDB code 1JLM) to GPIbα (residues 1-267; PDB code 1P9A) was performed with SWARMDOCK server web (version 15.04.01) (https://bmm.crick.ac.uk/∼svc-bmm-swarmdock/submit.cgi) (Jain, 2003). In docking, seven residues, four (T211, T213, K244, and R216) on Mac-1 and others (R218, R222, and N223) on GPIbα, were designated as binding site residues because of the mutation data (Simon et al., 2000; Wang et al., 2005). The N- and C-terminal in either of Mac-1 and GPIbα was set to be neutral. All docking results (444 complex structures) were grouped into ten clusters, in which each was defined as an ensemble of at least two complex models with ligand interface C_α_ RMSD <6 Å. The docking model with the lowest binding energy and the specified essential residues participating in the receptor–ligand interaction was considered as the best one. Each complex model was visually inspected by visual molecular dynamics (VMD), and only one was selected as the best model by the following criteria: the N- and C-terminal of Mac-1 could not be bound with the LRR domain of GPIbα because of the binding of the Mac-1 legs to both the N- and the C-terminus, and the model had not only the largest number of designated interface residues but also the lowest SWARMDOCK score. The best complex model, the so-called Model I, was selected from docking results and used for subsequent analysis.

### System Setup and Equilibrium

We herein used two software packages, VMD for visualization and modeling (Humphrey et al., 1996) and the NAMD 2.13 program for molecular dynamics simulations (Phillips et al., 2005). The Model I was solvated with TIP3P water molecules in a rectangular box (6.54 nm × 11.6 nm × 8.4 nm). The system was neutralized by adding 118 Na^+^ and 126 Cl^−^ (150 mM concentration) to mimic the actual physiological environment and consisted of 103,164 atoms. MD simulations were performed with periodic boundary condition and 2 fs time step as well as the CHARMM27 all-atom force field (MacKerell et al., 1998), along with cMAP correction for backbone, particle mesh Ewald (PME) algorithm for electrostatic interaction, a 12 Å cutoff for electrostatic, and Van der Waals interaction. All bonds were restrained using SHAKE to allow the time step of 2 fs. The system was energy minimized first for 15,000 steps with heavy or non-hydrogen protein atoms being fixed, and then for another 15,000 steps with all atoms free. The energy-minimized systems were heated gradually from 0 to 310 K in 0.1 ns first, and then equilibrated once for 100 ns with pressure and temperature control. The temperature was held at 310°K using Langevin dynamics, and the pressure was held at 1 atmosphere by the Langevin piston method. The equilibrated structure of Model I with better thermal stabilization was used as the initial conformation for the subsequent steered molecular dynamics (SMD) simulations (Supplementary Figure S1).

### Steered Molecular Dynamics Simulation

The SMD simulations in “force-ramp,” “force-ramp + snapback,” and “ramp + clamp” modes were performed for testing the mechanical strength, optimizing the structure of Model I, and the mechano-regulated structure–function interaction of the complex of Mac-1 with GPIbα, respectively. In the force-ramp MD simulation, the N-terminal C_α_ atom of GPIbα was fixed, and the C-terminal C_α_ atom of Mac-1 was pulled with constant pulling velocity (3 nm/ns) along the line between the steered and fixed atom (Supplementary Figure S1C) (Simon, 2012). The dummy atom and the steered atom were linked by the virtual spring with a spring constant of 13.89 pN/Å. The rupture force of the complex was read from the peak in the force–time pattern simulated with the force-ramp mode and used to scale the mechanical strength of the complex.

It is assumed that a rational docking model for the Mac-1/GPIbα complex should have both, a better thermal stabilization and stronger mechanical strength. To making Model I be more rational, we here developed a computer strategy of docking model optimizing (DMO) *via* the so-called force-ramp + snapback SMD simulations because the poor mechanical strength of the complex Model I was demonstrated by its low complex rupture forces. In a run with the “force-ramp + snapback” mode, an SMD simulation of 5 ns with constant pulling velocity (3 nm/ns) was performed first, then the system was mechanically unloaded but followed with equilibrium of 100 ns or 40 ns for the snapback complex. Through MD simulation with one or several mechanically loading–unloading cycles, as described above, the Model I might be remodeled and optimized as a more rational model, named Model II or Model III, for its better structural stabilization.

The so-called ramp-clamp SMD simulations, a force-clamp MD simulation followed a force-ramp one, were performed thrice on the equilibrated system with the Model II of the complex to examine the force-induced unbinding and conformation changing of the GPIbα bound with Mac-1. For each simulation, the complex was first pulled until the tensile force arrived at a given value, such as 25, 50, or 75 pN, and then, the SMD simulation was transformed from the force-ramp mode to a force-clamp one, at which the complex was stretched with the given constant tensile force for the following 40 ns.

### Data Analysis for MD Simulations

All analyses were treated with VMD tools. The C_α_ root mean square deviation (RMSD) and the solvent accessible surface area (SASA) (with a 1.4 Å probe radius) were used to characterize the conformational change and the hydrophobic core exposure, respectively. The binding energy, consisting of van der Waals energy and electrostatic energy, was calculated through the NAMD energy plugin in VMD. A hydrogen bond was formed if the donor–acceptor distance and the donor-hydrogen–acceptor angle were less than 3.5 Å and 30°, respectively. A salt bridge was built up once the distance between any of the oxygen atoms of acidic residues (Asp or Glu) and the nitrogen atoms of basic residues (Lys or Arg) was within 4 Å. An occupancy (or survival rate) of an H-bond or a salt bridge was scaled with the percentage of bond survival time in the simulation period. As a reflection of the mechanical strength of receptor–ligand complex (Grubmüller et al., 1996), the rupture force was read from the maximum of the force spectrum in a force-ramp run with constant pulling velocity. All visual inspections and molecular images were completed by using VMD. The formation or breakage of each hydrogen bond on the binding site was assumed to be an independent event not related to other bonds.

As a scale for the residue–residue interactions across binding site, $p_{ij}$, the probability of the *i*th ligand residue binding with the *j*th receptor residue, was evaluated by the following equation:

$$p_{ij}=1-\overset{M_{ij}}{\underset{l=1}{∐}}\left(1-\omega_{ij,l}\right),\;\;i=1,2,\ldots ,M_{L};j=1,2,\ldots ,M_{R};\;l=0,1,\ldots ,M_{ij},$$ (1)

where $\omega_{ij,l}$ was the survival ratio of the *l*th H-bond between the *i*th ligand residue and the *j*th receptor residue, $M_{ij}\left(\geq 0\right)$ denoted the numbers of H-bonds between the *i*th ligand residue and the *j*th receptor residue, and $M_{L}\left(\geq 1\right)$ and $M_{R}\left(\geq 1\right)$ were, respectively, the total numbers of ligand and receptor residues involved in binding. Thus, $P_{j,L}$ (the probabilities of the *j*th ligand residue binding to the receptor) and $P_{j,R}$ (the probabilities of the *j*th receptor residue binding to the ligand) were, respectively, estimated by the following equations:

$$P_{j,L}=1-\prod_{i=1}^{M_{R}}\left(1-p_{ji}\right)$$ (2)

and

$$P_{j,R}=1-\prod_{i=1}^{M_{L}}\left(1-p_{ij}\right).$$ (3)

Furthermore, $P_{D}$, the dissociation of ligand from receptor, could be estimated by the following equation:

$$P_{D}=1-\prod_{j=1}^{M_{L}}\left(1-P_{j,L}\right)=\;1-\prod_{j=1}^{M_{R}}\left(1-P_{j,R}\right).$$ (4)

And, the mechano-regulation factor or the normalized complex dissociation $f_{D}$ was the ratio of $P_{D}$ at tensile force of $f_{0}$ and of $P_{D}$ at zero tensile force, that was given by the following equation:

$$f_{D}=P_{D}{\left|\frac{f=f_{0}}{P_{D}}\right|}_{f=0},$$ (5)

where *f* expressed the tensile force on the complex and *f*_0_ was a given tensile force. Regardless of the geometrical and timescale effects on complex dissociation $P_{D}$, it was expected that $f_{D}$ should be comparable with our AFM experiment data.

## Results

### A Likely Wild-Type Model of Mac-1–GPIbα Complex Was Well Built Up Through Molecular Docking With Treatment of the “Force-Ramp + Snapback” MD Simulations

**FIGURE 1:**
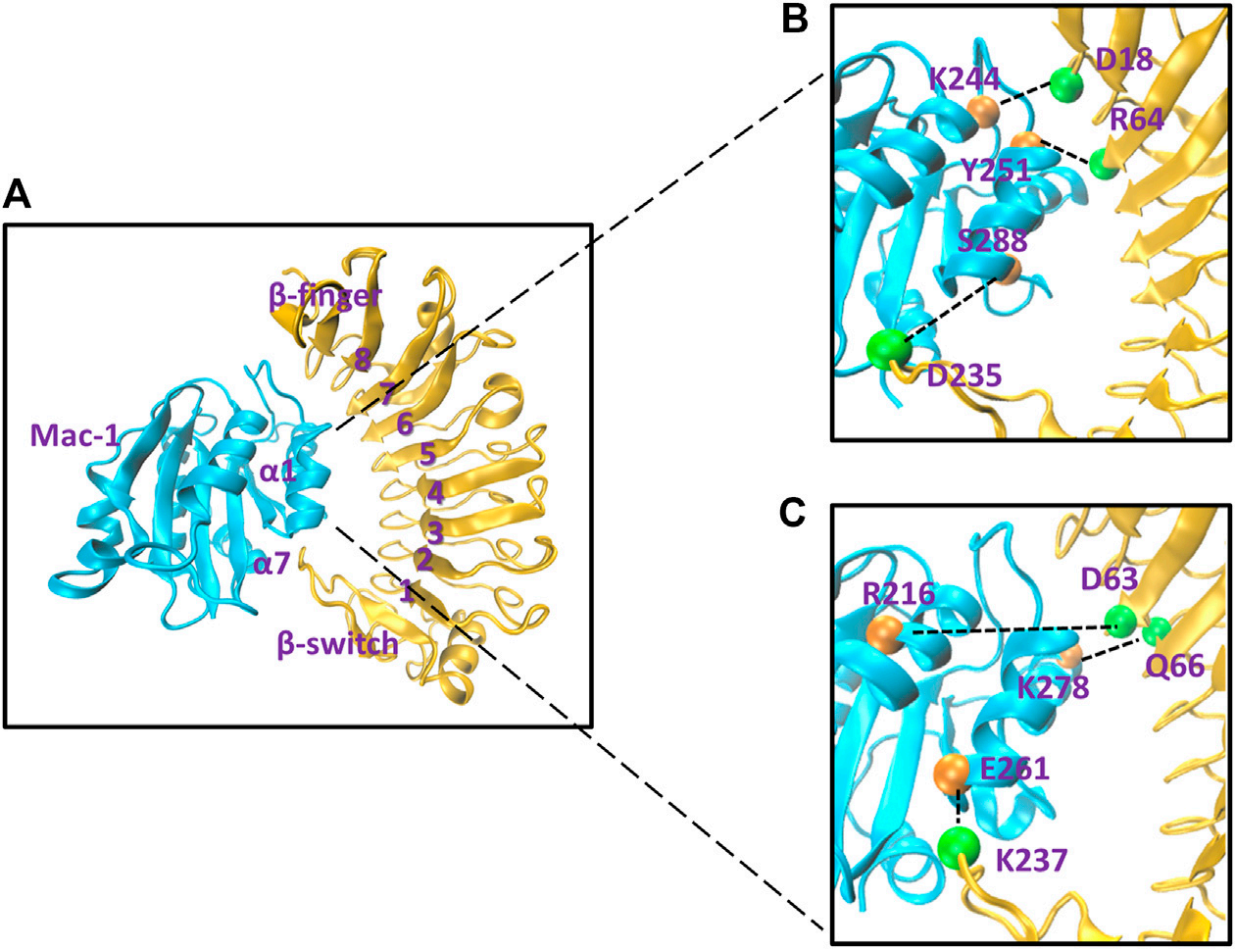
The likely wild-type molecular docking model and some representative involved hydrogen bonds on the binding site of the Mac-1/GPIbα complex. **(A)** The snapshot of the likely wild-type molecular docking model, which was built up by treating Model I of the Mac-1/GPIbα complex with a “ramp-snapback” MD simulation (Material and Methods) and shown in new cartoon diagram. Mac-1 and GPIbα were colored with cyan and orange, respectively. The hydrophobic pocket of GPIbα consists of six β sheets (from β1 to β6), seven α helixes (from α1 to α7), and loops to link any two adjacent α or β structures. **(B)** The three intrinsic hydrogen bonds, which were contributed by the residue pairs such as K244-D18 and Y51-R64 as well as S288-D235 and detected from either of Model I and II. **(C)** The three newly formed hydrogen bonds, which were the linkers between the other three residues (R216-D63, K278-Q66, and E261-237) and occurred just at Model II. The hydrogen bonds at the complex interface were shown as dashed black lines and labeled on the structure in VDW mode. The orange spheres represent the residue on Mac-1 and the green spheres represent the residue on GPIbα.The labels here see reference (Kobe and Kajava, 2001).

To gain a likely wild-type conformation for the complex of Mac-1 with GPIbα, we built three structural models (Models I, II, and III) for complex of Mac-1 with GPIbα, through SWARMDOCK program (Torchala et al., 2013) with and without a DMO treatment (Materials and Methods), respectively. With the lowest SWARMDOCK energy score and the most mutation-identified residues in the binding site, the Model I was picked out from 444 poses generated by docking of Mac-1 to ligand-free GPIbα and equilibrated by performing a system equilibrium of 100 ns or 40 ns along with the same protocol of energy minimization (see Material and Methods). Models II and III (Figure 1A) were built up, respectively, by remodeling Model I with the so-called force-ramp + snapback SMD simulation of 105 ns or 45 ns, in which 5 ns was spent for the SMD simulation with a pulling velocity of 3 nm/ns, and the other 100 ns or 40 ns was contributed to a system re-equilibration for the unloaded or snapped-back complex (Materials and Methods). Models I, II, and III should be equilibrated because the time courses of the total energy, and the root mean square deviation (RMSD) of C_α_-atoms fluctuated on their respective stable levels with small relative derivations (Supplementary Figure S2A,B,E). Among the all fourteen observed H-bonds across the complex interface in Model II (Table 1; Supplementary Table S1), the first seven existed also in Model I, but others did not (Figures 1B,C); and except the 7th bond, the other six in Model I had lower occupancies than those in Models II and III. It suggested that the missed or undervalued H-bonding events on the interface in Model I might emerge and be valuation-rational through treatment with “force-ramp + snapback” SMD simulation.

**TABLE 1:** Hydrogen bonds on the binding site of the complex.

No	Residue pair		Occupancy		
	Mac-1	GPIbα	Model I	Model II	Model III
1	K244	D18	0.39	0.77	0.74
2	E282	K19	0.62	0.66	0.67
3	S288	D235	0.23	0.52	0.57
4	E242	K19	0.35	0.44	0.43
5	E252	S39	0.31	0.44	0.53
6	Y251	R64	0.17	0.34	0.53
7	K278	E40	0.67	0.32	0.28
8	E261	K237	0	0.54	0.59
9	R216	D63	0	0.31	0.25
10	K278	Q66	0	0.27	0.23
11	D259	K231	0	0.23	0.15
12	K278	R64	0	0.16	0.12
13	H294	K231	0	0.16	0.2
14	K289	K231	0	0.16	0.18

**FIGURE 2:**
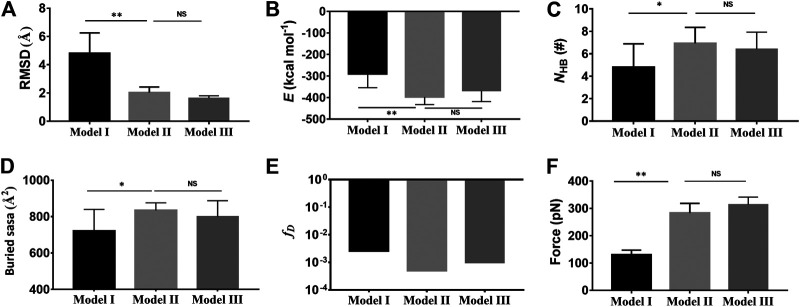
Comparison of the complex characters in Model II (light gray) and Model III (dark gray) with those in Model I (black). **(A)** The mean C_α_-RMSD, **(B)** the mean binding energy *E* (−293 ± 75 kcal/mol for Model I, -398 ± 53 kcal/mol for the II, and −369 ± 50 kcal/mol for Model III), **(C)** the mean interface H-bond number *N*_HB_ (4.8 ± 2 for Model I, 6.9 ± 1.4 for Model II, and 5.8 ± 1.5 for Model III), **(D)** the mean buried SASA (730 Å^2^ for Model I, 840 Å^2^ for Model II, and 800 Å^2^ for Model III), and **(E)** the dissociation probability *f*_D_ for complex in 40 ns equilibrium. **(F)** The mean rupture force (120 pN about for Model I, 300 pN for Model II, and 313 pN for Model III) in “force-ramp” SMD simulations thrice on complex with a velocity of 3 Å/ns. The *p*-values of the unpaired two-tailed Student’s t test were shown to indicate the statistical difference significance (****p < 0.0001), or lack thereof. Data were shown with mean ± S.D.

The mean C_α_-RMSD, binding energy (*E*), the interface H-bond number (*N*_HB_), and the interface buried SASA for complex in equilibrium of 40 ns showed that the C_α_-RMSD value climbed from 2 Å to a quasi-plateau of 6 Å for the Model I but remained almost at a low level of 2 Å for Models II and III (Figure 2A; Supplementary Figure S2B), suggesting a higher thermo-stabilization of Models II and III in comparison with Model I (Figure 2A); Models II and III rather than Model I should be energy favorable because the binding energies (−398 ± 53 kcal/mol, −369 ± 50 kcal/mol) in Models II and III were far lower than that (−293 ± 75 kcal/mol) in Model I (Figure 2B); the mean number of H-bonds on the binding site over a simulation time of 40 ns for Models I, II, and III were 4.8, 6.9, and 5.8 (Figure 2C), respectively, showing a stable linkage between Mac-1 and GPIbα for Models II and III rather than Model I; and the mean interface buried SASA was read to be 730 Å^2^ for Model I, 840 Å^2^ for Model II, and 800 Å^2^ for Model III (Figure 2D), meaning the closer contact between Mac-1 and GPIbα in Models II and III than that in Model I. The dissociation probabilities $\left(f_{D}\right)$ of complex were estimated to be 0.02, 0.0005, and 0.0009 for Models I, II, and III (Figure 2E), showing that the Mac-1 affinity to GPIbα for the Model I was down estimated and could be restored to the quasi-actual level by a DMO treatment based on a “force-ramp + snapback” MD simulation. These results demonstrated that in comparison with Model I, Models II and III were more energy-rational and more thermostable. And, we further performed the so called force-ramp SMD simulations thrice with constant pull velocity (3 nm/s) for Models I, II, and III (Materials and Methods) to evaluate the mechanical strength of the models. The force–time curves exhibited that the rupture force of the complex was 150 pN about for Model I but 300 pN about for Models II and III, suggesting a high mechano-strength in Models II and III rather than in Model I (Figure 2F; Supplementary Figure S2E). Under pulling with a constant velocity of 3 Å/ns, the Mac-1/GPIbα complex remained structure-stable under a pulling force <250 pN for Models II and III or 100 pN for Model I (Supplementary Figure S2E; Supplementary Videos S1, S2). These results demonstrated that in comparison with Model I, Models II and III were more energy-rational, more thermo- and mechano-stable in modeling the Mac-1/GPIbα complex. For these reasons, Model II was regarded as the likely wild-type model of the Mac-1/GPIbα complex and used as an initial conformation for the subsequent “ramp-clamp” SMD simulations.

### Dissociation of the Stretched Mac-1–GPIbα Complex Was Biphasic Force Dependent

**FIGURE 3:**
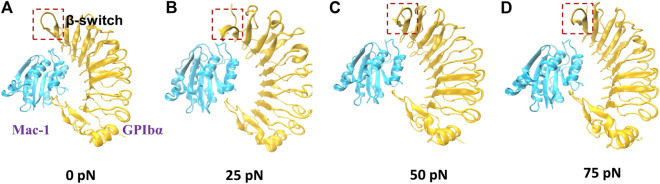
The conformations of Mac-1/GPIbα complex (Model II; new cartoon) after 40 ns “clamp-force” SMD simulation with tensile forces 0 **(A)**, 25 **(B)**, 50 **(C)**, and 75 pN **(D)**. Mac-1 and GPIbα were shown as cyan and orange, respectively. The main conformation changes of β-switch, which were marked with a red box, where shown in **(A–D)**.

**FIGURE 4:**
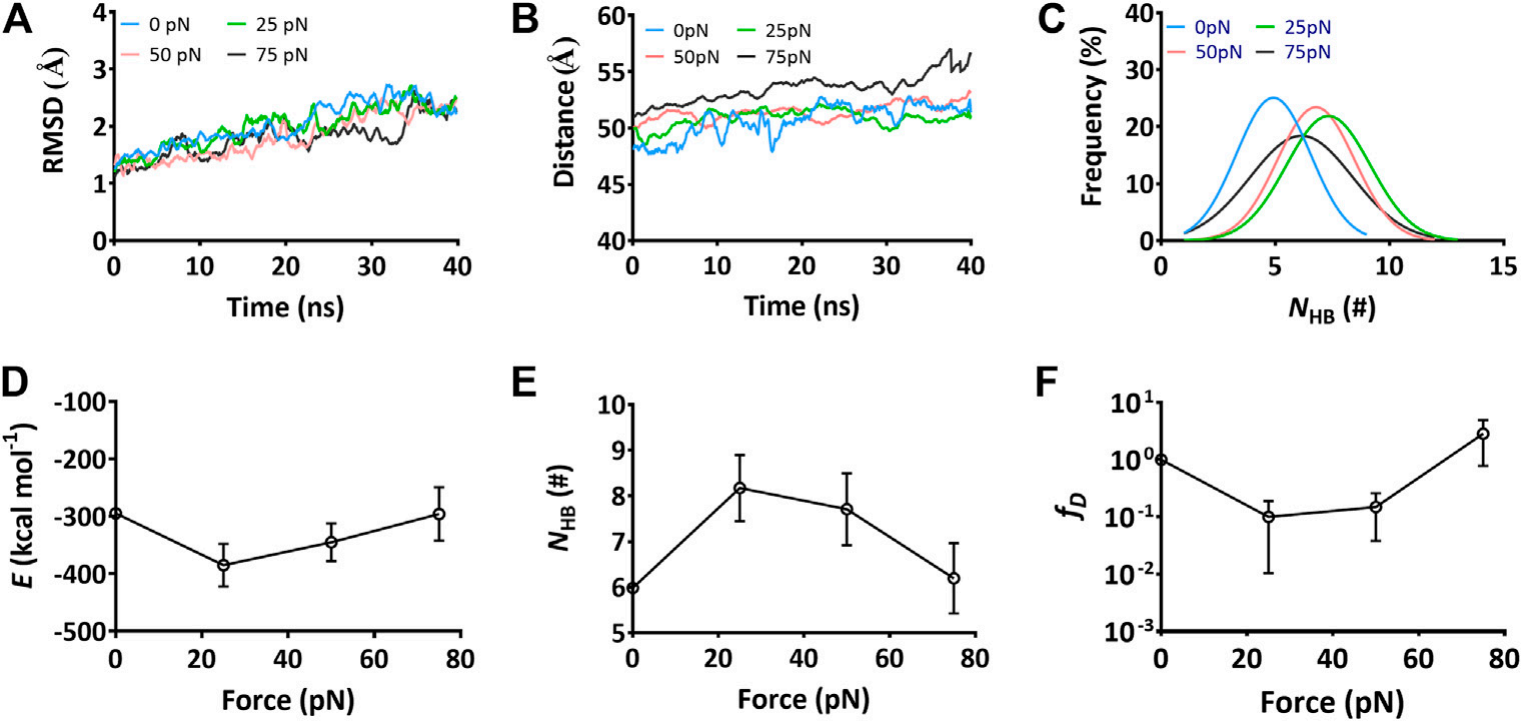
Variations of the structural stability and interface interaction of the complex versus constant tensile force. Data were read from the thrice 40-ns “force-clamp” SMD simulations on Model II of the Mac-1–GPIbα complex. **(A)** The representative time courses of the C_α_-RMSD of complex at tensile forces of 0 (blue), 25 (green), 50 (red), and 75 pN (black). The C_α_-RMSD fluctuated in a range from 1.0 to 2.5 Å for each tensile force. **(B)** The distance–time plot at tensile forces of 0, 25, 50, and 75 pN. The distance between the pulled- and fixed-atom fluctuated with an amplitude <5Å around a plateau for each tensile force. **(C)** Gaussian fitting of the N_HB_ frequencies from thrice 40-ns runs at various tensile forces. **(D)** and **(E)** The variations of the mean binding energy *E* and the mean H-bond number *N*_HB_ on binding site over 40 ns for three runs versus the tensile force. **(F)** The normalized dissociation probability *f*_D_ of complex under various tensile forces. Pearson correlation coefficients for *E*, *N*_HB_, and *f*_D_ are -0.832, 0.879, and -0.987 if 0 ≤ force ≤ 25 pN but take values of 0.595,−0.766, and 0.749 as 25 pN < force ≤ 75 pN, respectively, with *p* < 0.05, statistically demonstrating the dependences of *E*, *N*_HB_, and *f*_D_ on the tensile force. The data in **(D)**, **(E)**, and **(F)** were shown as the mean ± SD.

To examine the mechano-regulation on the interaction of Mac-1 with GPIbα, we performed a series of “ramp-clamp” SMD simulations of 40 ns thrice with Model II under constant tensile forces of 0, 25, 50, and 75 pN (Materials and Method). Just a bit force-induced conformational change of complex is shown in Figure 3, meaning that Model II was reliable for its fine mechanical stability. And, the mechanical stability of the complex was also demonstrated by the very slight tension-induced increasing of the C_α_-RMSD of the complex (Figure 4A) and distance between the pulled- and fixed-atom (Figure 4B), while the sampled structural space was regarded as quasi-perfect because the H-bond number obeyed the Gaussian distribution (Figure 4C), meaning that the complex conformations sampled within a simulation time of 40 ns under each given constant tensile force were enough in gaining information of the structure–function relation of the complex.

**FIGURE 5:**
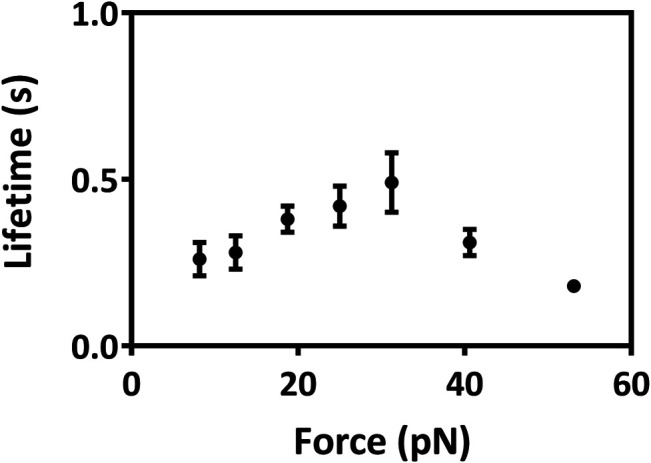
Variation of lifetimes of Mac-1–GPIbα bonds versus force. All data were from single molecular AFM measurements and shown as mean ± SEM. At least 325 single-bond rupture force data at each group were collected and analyzed using a force bin of 7.5 pN. Cantilever tip and petri dish were functionalized through coating with 15 μg/ml Mac-1 and GPIbα, respectively (Materials and Methods).

The interaction energies, the buried SASA, and the H-bonds (or salt bridges) on the binding site for the complex under constant tensile forces were sampled through the “ramp-clamp” SMD simulations with Model II (Materials and Method). Plots of the mean interaction energy (*E*), the mean buried SASA, and the mean number of the H-bonds (*N*_HB_) (or salt bridges) on the binding site over 40 ns for three runs against tensile force exhibited that *E* decreased first and then increased with *F*, and the force threshold occurred at 25 pN (Figure 4D), demonstrating a biphasic force-dependent energy preference for the stretched GPIbα–Mac-1 complex; on the contrary, *N*_HB_ increased first and then decreased with *F* (Figure 4E), illustrating a transition from force-enhanced to force-weakened linkage between GPIbα and Mac-1; as a result, *f*_D_, the normalized complex dissociation probability, decreased first and then increased *F* (Figure 4F), suggesting a catch-slip bond transition in Mac-1 dissociation from GPIbα. All the transition points for *E*, *N*_HB_, *f*_D_, and the mean buried SASA occurred at a tensile force of 25 pN, as it should be. These results were in keeping with our single molecular AFM measurement data, which exhibited a catch-slip bond transition with a force threshold of about 31 pN (Figure 5; Supplementary Figure S3) for interaction between Mac-1 with GPIbα. The catch-slip bond transition had been measured by AFM and BFP as well as flow chamber experiments for various adhesive molecule systems, such as von Willebrand factor with GPIbα (Yago et al., 2008), ADMAMTS13 (Wu et al., 2010), PSGL-1 with P-, E-, and L-selectins as well as the PSGL-1-actin cytoskeleton linker protein ezrin/radixin/myosin (ERM) (Marshall et al., 2003; Yago et al., 2004; Li et al., 2016), and so on.

It signed that the computer strategy with “ramp-clamp” SMD simulation was practicable in examining the mechano-regulation on receptor–ligand interactions, as done in our previous work for the interaction of PSGL-1 with ERM (Feng et al., 2020) or Kindlin 2 with β_3_ integrin (Zhang et al., 2020) and Model II, a docking model treated with “force-ramp + snapback” SMD simulation, was suitable in studying the structure–function relation for the complex of Mac-1 with GPIbα.

### Force-Induced Allostery in Mac-1 Dissociation From GPIbα

**FIGURE 6:**
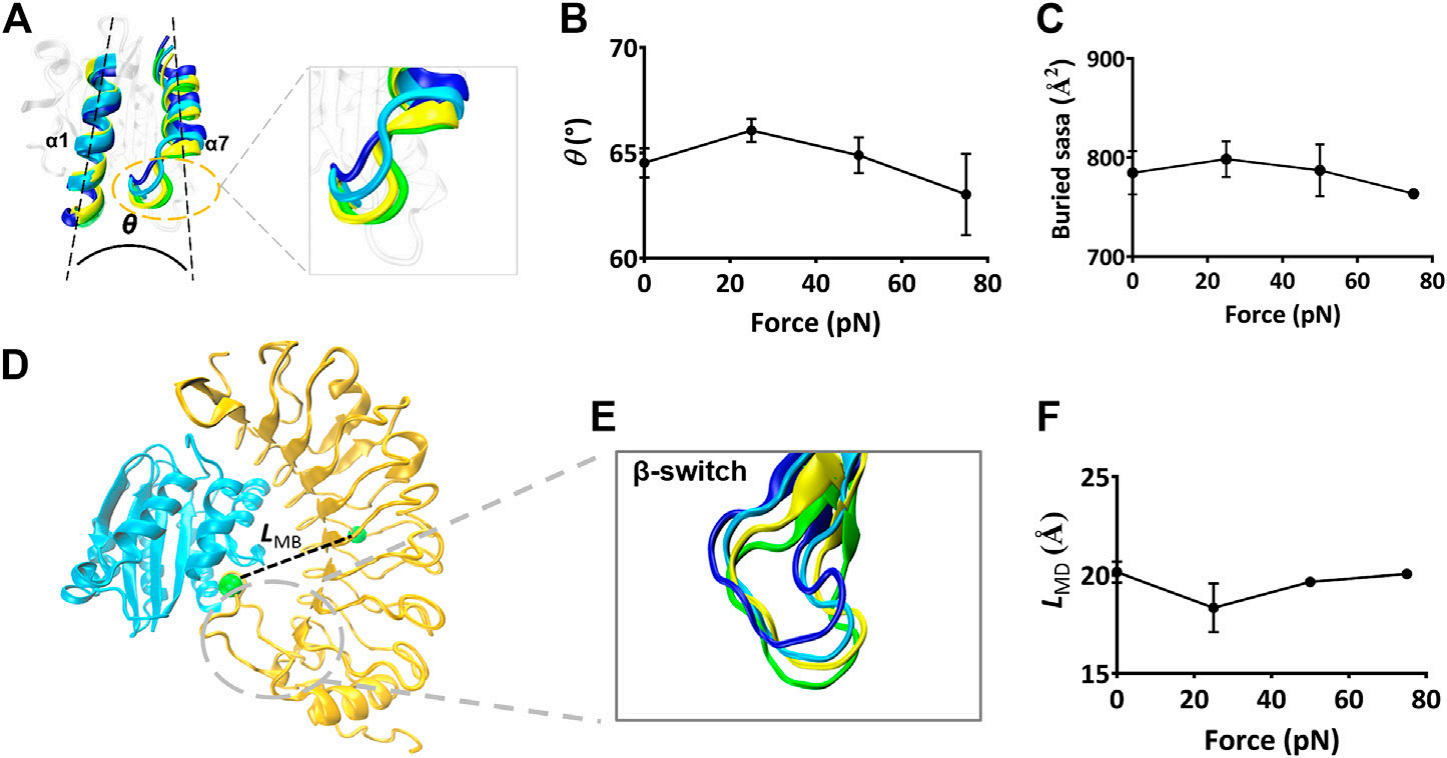
The force-induced conformational change of the Mac-1–GPIbα complex. **(A)** The cross angle *θ* between α7- and α1-helix of the ligated Mac-1. The four representative conformations of α1- and α7-helix under various tensile forces of 0, 25, 50, and 75 pN were colored in blue, cyan, yellow, and green, respectively. The superposition of these different conformations showed a force-induced down-movement of α7 helix tail. **(B)** The plot of *θ* against tensile force. *θ* was averaged over the simulation time of 40 ns for three runs. The mean values are 64.5, 66.5, 64.4, and 62.3 degrees, respectively. **(C)** Variation of the mean buried SASA over the thrice 40-ns runs versus tensile force. **(D)** The distance *L*_MB_ (dashed black line) between the two green spheres, which were located respectively at the centroid and the D235 residue in the bound GPIbα. **(E)** Superposition of four representative conformations of β-switch (residues 227–241) of GPIbα under various tensile force of 0 (blue), 25 (cyan), 50 (yellow), and 75 pN (green). A force-induced down-movement of the β-switch was shown in the structural superposition. **(F)** Variation of LMB versus the tensile force. Pearson correlation coefficients for *θ*, SASA, and *L*_MB_ are 0.852, 0.408, and −0.735 if 0 ≤ force ≤ 25 pN but take values of −0.606, −0.612, and 0.885 as 25 pN < force ≤75 pN, respectively, with *p* < 0.05, statistically demonstrating the dependences of *E*, *N*_HB_, and *f*_D_ on the tensile force. The data in **(B)**, **(C)**, and **(F)** were shown as the mean ± SD.

To scale the force-induced allostery of the Mac-1 bound with GPIbα, we herein measured *θ* (Figure 6A), the mean angle between α1 and α7 helix of the ligated Mac-1 over 40 ns simulation time thrice under each tensile force. The angle *θ* increased remarkably first and then decreased with *F* (Figure 6B), was correlative negatively to the normalized complex dissociation probability *f*_D_ (Figure 4F) and the interaction energy *E* (Figure 4D) but positively to the H-bond number *N*_HB_ (Figure 4E) and the mean buried SASA (Figure 6C), suggesting that the force-induced allostery of the ligated Mac-1 might be responsible for the catch-slip bond transition in the interaction of Mac-1 with GPIbα. An observation for the α7 helix of Mac-1 exhibited a descent of Mac-1 affinity to GPIbα due to the downward change of the α7 helix (Figure 6A).

Besides, we measured *L*_MB_, the distance from the C_α_ atom of the residue D235 in the β-switch to the mass center of GPIbα (Figure 6D), to scale the deviation of the β-switch from its neighbor subdomains under various tensile forces. Plots of *L*_MB_ against tensile force (Figure 6F) said that increasing tensile force made *L*_MB_ lengthened significantly first and then shortened slightly, and the turning point occurred at the tensile force of 25 pN too, demonstrating that a limit on Mac-1 dissociation from GPIbα might be provided through the β-switch deviating from GPIbα body. Together with the force-induced allostery of the ligated Mac-1 (Figures 6A,B), the force-mediated deviation of the β-switch (Figure 6E) also might be responsible for the force-dependent mean buried SASA of the binding sites and the dissociation of Mac-1 from GPIbα.

### The Key Residues in the Biphasic Force-Dependent Mac-1-GPIbα Interaction

To reveal the structural basis of the biphasic force-dependent Mac-1–GPIbα interaction mentioned above, we examined the H-bonding interactions on the binding site through “force-clamp” SMD simulation of 40 ns thrice under tensile forces of 0, 25, 50, and 75 pN, and evaluated the probabilities for unbinding of either the residues in Mac-1 from GPIbα or the residues in GPIbα from Mac-1 (Materials and Methods). The variation of the H-bond occupancy versus tensile force (Table 2) demonstrated that the residue–residue interactions on the binding site were mechano-sensitive, and increasing tensile force might make H-bonding occurred, broken, strong, or week, exhibiting a diversity for the H-bonds in response to the tensile force. Of all detected H-bonds, those with mean occupancies >0.20 had eleven members (Table 2; Supplementary Table S2) which could be clustered into four groups in responding to the tensile force with modes of the “slip-bond type,” the “catch-slip bond” type, the “slip-catch-slip bond,” and the “catch-slip-catch bond” type, respectively. The first group was contributed by K19 on GPIbα with its two partners E243 and E282 on Mac-1; the second was consisted of those such as D235 on GPIbα paired with S288, K278, and E261 on Mac-1 paired with their respective partners E40 and K237 on GPIbα, as well as E252 on Mac-1 with its three partners K37, S39, and R64 on GPIbα; the third included those of K244 and Y251 on Mac-1 paired with their respective partners, D18 and R64 on GPIbα; and the fourth was contributed only by D259 on Mac-1 paired with K231 on GPIbα (Table 2). These suggested that the force-induced changes of conformation and function for either Mac-1 or GPIbα in complex should be mediated by the cooperative interaction of the H-bonds in responding to the tensile force with different modes.

**TABLE 2:** H-bonds (with occupancies in top 11) on the binding site of the complex under various tensile forces.

No	Residue Pair		Occupancy			
	Mac 1	GPIbα	0 (pN)	25 (pN)	50 (pN)	75 (pN)
1	E252	K37	0.08	0.19 ± 0.11	0.43 ± 0.15	0.03 ± 0.009
2	E252	S39	0.44	0.67 ± 0.12	0.83 ± 0.03	0.39 ± 0.18
3	E252	R64	0.10	0.74 ± 0.03	0.48 ± 0.23	0.49 ± 0.20
4	E261	K237	0.54	0.58 ± 0.01	0.57 ± 0.03	0.39 ± 0.19
5	K278	E40	0.32	0.62 ± 0.04	0.55 ± 0.08	0.56 ± 0.007
6	S288	D235	0.52	0.79 ± 0.08	0.77 ± 0.04	0.62 ± 0.05
7	K244	D18	0.77	0.61 ± 0.14	0.66 ± 0.03	0.58 ± 0.14
8	Y251	R64	0.34	0.14 ± 0.03	0.28 ± 0.08	0.16 ± 0.09
9	D259	K231	0.23	0.48 ± 0.03	0.25 ± 0.12	0.33 ± 0.16
10	E243	K19	0.44	0.37 ± 0.08	0.24 ± 0.14	0.19 ± 0.08
11	E282	K19	0.66	0.64 ± 0.06	0.47 ± 0.1	0.48 ± 0.13

Based on the contributions on strong interface H-bonding with high occupancies (>50%), the six residues, such as K244, E252, E261, K278, E282, and S288 on Mac-1, might be responsible for the force-dependent interaction of Mac-1 with GPIbα, despite just K244 in these residues was mutation-identified (Simon et al., 2000; Wang et al., 2005). Other three mutation-identified residues, such as T211 and T213 as well as R216 on Mac-1 (Simon et al., 2000; Wang et al., 2005), did not emerge from the above six key interface residues because of either nothing for T211 and T213 or less contribution for R216 to H-bonding on the complex interface. This inconsistency of the mutation-experimental data and the computational results might come from the timescale effects on milliseconds MD simulations in predicting the receptor–ligand interaction in a period of 1 s or its tenth (Schwantes et al., 2014).

## Discussion

It was believed that MD simulation might predict druggable binding site on complex interface, while providing a valuable dynamics insight to receptor–ligand interaction mechanism. However, a near-native docking model should be required for a meaningful MD simulation, under the lack of solved structural data of the complex. A rational assumption said that Mac-1/GPIbα complex should be thermo- and mechano-stable, like the GPIbα–VWF complex because of the structural similarity between the two complexes. However, it is still a technical challenge to make a complex docking model near-native. We herein proposed a novel computer strategy to make the Model I (the docking model of Mac-1/GPIbα complex) more near-native or rational. This strategy for structural improvement included a system equilibrium followed a “force-ramp + snapback” SMD simulation of 105 ns, in which 5 ns was spent for SMD simulation with a constant pulling velocity of 3 nm/ns and the other 100 ns for a system re-equilibration for the unloaded or snapped-back complex (Materials and Methods). The interface H-bonds in Model II were more and stronger than those in Model I, saying that the present computer strategy with a treatment of “force-ramp + snapback” MD simulation might be feasible for improving a docking model, and meaning that the barrier in the transition from a nonnative complex conformational model to a near-native one might overcome through adding a mechano-perturbation on the complex. However, the effectiveness and efficiency of our “force-ramp + snapback” methodology relies on a suitable choice of fixed and pulled atoms, as well as pulling velocity and direction. We have shown that given a rational choice of these parameters, our methodology can improve the quality of a modeled dimer. To conclusively demonstrate the general applicability of our methodology, tests on multiple docked models of different dimers should be carried out.

As a key event in hemostasis and inflammatory responses to vessel injuries, the crosstalk between platelet and neutrophil would be mediated by GPIbα–Mac-1 interaction in hemodynamic environments. Lack of crystal structural data led to less knowledge on the mechano-regulation and its molecular basis on GPIbα–Mac-1 interaction under shear stress conditions, despite those mutation-identified residues, such as T211, T213, K244, and R216 on Mac-1, were demonstrated to be crucial for binding of Mac-1 to GPIbα (Simon et al., 2000; Wang et al., 2005). In mediating adhesion and accumulation of circulating platelets, GPIbα–vWF interaction was governed by a catch-slip bond mechanism, saying a force-induced prolongation of bond lifetime for complex under loads below a force threshold (Da et al., 2014). This catch-slip bond was also observed herein not only from AFM measurements for GPIbα–Mac-1 interaction but also from a series of “ramp-clamp” mode SMD simulations with Model II of the GPIbα–Mac-1 complex under various tensile forces (Figure 4). It might come from the structural similarity between Mac-1 I-domain and VWF-A1 domain with the major binding site for GPIbα (Diamond et al., 1993). This better consistency of the experimental data and computational predictions might provide another support to Model II of the GPIbα–Mac-1 complex, despite that the catch-slip bond transition occurred at 31 pN in AFM experiments but 25 pN in the “ramp-clamp” SMD simulations. The catch-slip bond phenomenon in GPIbα–Mac-1 interaction was observed in various adhesive molecular systems, such as selectins with PSGL-1 (Marshall et al., 2003), β_2_ integrin (α_L_β_2_, α_M_β_2_) with ICAM-1 (Kong et al., 2009; Chen et al., 2011), and VWF-A1 with GPIbα (Yago et al., 2008; Lining et al., 2015).

The force-induced allostery of the mutually constrained Mac-1 and GPIbα was stable for a given tensile force (Figure 3) and might synergize beneficially to induce the “catch-slip bond” phenomenon in the interaction of Mac-1 and GPIbα. We obtained that the catch bond in the interaction of Mac-1 to GPIbα might be derived from an increasing flexibility of the αM domain α1 helix and a force-induced downward movement of the αM domain α7 helix of the bound Mac-1, similar to the α7 helix shifting downward and the outward movement of the α1 helix in the force-induced conformational transition of the ICAM-1–bound LFA-1 (Chen et al., 2011). The force-induced change of angle between α1 and α7 helix came from the swing of α7 tail spiral (Figures 6A,B), suggesting that α7 helix was responsible for the affinity of the bound Mac-1. The force-induced change of the GPIbα-binding pocket (the β-switch) might regulate the GPIbα affinity to Mac-1 (Figures 6D–F), in consistency with the interaction of VWF A1 domain with GPIbα.

Usually, MD simulation results at the atom level were not comparable to the single molecular measurement data. The barriers might mainly come from the timescale effects on MD simulation results in predicting receptor–ligand interactions, due to that affinity change and conformation evolution of adhesive molecules would undergo a period far longer than the simulation timescale from nanoseconds to milliseconds (Schwantes et al., 2014). These timescale effects might be overcome through a suitable computer strategy such as the “ramp-clamp” SMD simulation, as shown in the better consistency of AFM experimental data with MD simulation results. With Model II of the Mac-1/GPIbα complex, the identified residues D222 and R218 on Mac-1 were predicted to be the key, showing the rationality of Model II and the availability of the present computer strategy. However, the random feature and the initial-state dependence of conformational evolution might lead to fail in detecting the identified residues (T211 and T213) on Mac-1 and N223 on GPIbα herein, but enough simulations in parallel might be beneficial in locating this residue.

In conclusion, a rational docking model (Model II) for the Mac-1/GPIbα complex was built herein through the present computer strategy, and shown to be thermo- and mechano-stable. A slip-catch bond transition phenomenon was observed not only from the “ramp-clamp” SMD simulations with Model II under various tensile forces but also from AFM experiments. The force-enhanced interaction of Mac-1 to GPIbα under force below a force threshold might be required for stable crosstalk between platelets and neutrophils in mechano-microenvironments around the injured vessel sides. The present work provided not only an effective computer strategy to build a likely wild-type model of Mac-1 bound to GPIbα but also a novel insight into the mechano-regulation mechanism and its molecular structure basis for Mac-1–GPIbα interaction and should be helpful for understanding the force-dependent platelet–leukocyte interactions in hemostasis and inflammatory responses under flows.

## Data Availability

The raw data supporting the conclusions of this article will be made available by the authors, without undue reservation.

## References

[B1] Behnen M. Leschczyk C. Moller S. Batel T. Klinger M. Solbach W. (2014). Immobilized immune complexes induce neutrophil extracellular trap release by human neutrophil granulocytes via FcγRIIIB and Mac-1. J. Immunol. 193 (4), 1954–1965. 10.4049/jimmunol.1400478 25024378

[B2] Chen W. Lou J. Zhu C. (2011). Forcing switch from short- to intermediate- and long-lived states of the alphaA domain generates LFA-1/ICAM-1 catch bonds. J. Biol. Chem. 285 (46), 35967–35978. 10.1074/jbc.M110.155770 PMC2975219 20819952

[B3] Coxon A. Rieu P. Barkalow F. J. Askari S. Sharpe A. H. von Andrian U. H. Arnaout M. A. (1996). A novel role for the beta 2 integrin CD11b/CD18 in neutrophil apoptosis: a homeostatic mechanism in inflammation. Immunity 5 (6), 653–666. 10.1016/s1074-7613(00)80278-2 8986723

[B4] Da Q. Behymer M. Correa J. I. Vijayan K. V. Cruz M. A. (2014). Platelet adhesion involves a novel interaction between vimentin and von Willebrand factor under high shear stress. Blood 123 (17), 2715–2721. 10.1182/blood-2013-10-530428 24642750 PMC3999756

[B5] Diacovo T. G. Roth S. J. Buccola J. M. Bainton D. F. Springer T. A. (1996). Neutrophil rolling, arrest, and transmigration across activated, surface-adherent platelets via sequential action of P-selectin and the beta 2-integrin CD11b/CD18. Blood 88 (1), 146–157. 10.1182/blood.v88.1.146.bloodjournal881146 8704169

[B6] Diamond M. S. Alon R. Parkos C. A. Quinn M. T. Springer T. A. (1995). Heparin is an adhesive ligand for the leukocyte integrin Mac-1 (CD11b/CD1). J. Cell Biol. 130 (6), 1473–1482. 10.1083/jcb.130.6.1473 7559767 PMC2120570

[B7] Diamond M. S. Garcia-Aguilar J. Bickford J. K. Bickford J. K. Corbi A. L. Springer T. A. (1993). The I domain is a major recognition site on the leukocyte integrin Mac-1 (CD11b/CD18) for four distinct adhesion ligands. J. Cell Biol. 120 (4), 1031–1043. 10.1083/jcb.120.4.1031 7679388 PMC2200080

[B8] Ehlers R. Ustinov V. Chen Z. Zhang X. Rao R. Luscinskas F. W. (2003). Targeting platelet-leukocyte interactions: identification of the integrin Mac-1 binding site for the platelet counter receptor glycoprotein Ibalpha. J. Exp. Med. 198 (7), 1077–1088. 10.1084/jem.20022181 14530377 PMC2194217

[B9] Fang X. Fang Y. Liu L. Liu G. Wu J. (2012). Mapping paratope on antithrombotic antibody 6B_4_ to epitope on platelet glycoprotein Ibalpha via molecular dynamic simulations. PLoS One 7 (7), e42263. 10.1371/journal.pone.0042263 22860101 PMC3408434

[B10] Fang X. Lin J. Fang Y. Wu J. (2018). Prediction of spacer-α6 complex: a novel insight into binding of ADAMTS13 with A2 domain of von Willebrand factor under forces. Sci. Rep. 8 (1), 5791. 10.1038/s41598-018-24212-6 29636514 PMC5893608

[B11] Feng J. Zhang Y. Li Q. Fang Y. Wu J. (2020). Biphasic force-regulated phosphorylation site exposure and unligation of ERM bound with PSGL-1: a novel insight into PSGL-1 signaling via steered molecular dynamics simulations. Int. J. Mol. Sci. 21 (19), 7064. 10.3390/ijms21197064 PMC7583015 32992803

[B12] Grubmüller H. Heymann B. Tavan P. (1996). Ligand binding: molecular mechanics calculation of the streptavidin-biotin rupture force. Science 271 (5251), 997–999. 10.1126/science.271.5251.997 8584939

[B13] Hidalgo A. Chang J. Jang J. E. Peired A. J. Chiang E. Y. Frenette P. S. (2009). Heterotypic interactions enabled by polarized neutrophil microdomains mediate thromboinflammatory injury. Nat. Med. 15 (4), 384–391. 10.1038/nm.1939 19305412 PMC2772164

[B14] Humphrey W. Dalke A. Schulten K. (1996). VMD: visual molecular dynamics. J. Mol. Graphics 14 (1), 33–38. 10.1016/0263-7855(96)00018-5 8744570

[B15] Jain A. N. (2003). Surflex: fully automatic flexible molecular docking using a molecular similarity-based search engine. J. Med. Chem. 46 (4), 499–511. 10.1021/jm020406h 12570372

[B16] Karshovska E. Weber C. Hundelshausen P. (2013). Platelet chemokines in health and disease. Thromb. Haemost. 110 (11), 894–902. 10.1160/TH13-04-0341 23783401

[B17] Kobe B. Kajava A. V. (2001). The leucine-rich repeat as a protein recognition motif. Curr. Opin. Struct. Biol. 11 (6), 725–732. 10.1016/s0959-440x(01)00266-4 11751054

[B18] Kong F. García A. J. Mould A. P. Humphries M. J. Zhu C. (2009). Demonstration of catch bonds between an integrin and its ligand. J. Cell Biol. 185 (7), 1275–1284. 10.1083/jcb.200810002 19564406 PMC2712956

[B19] Kruss S. Erpenbeck L. Amschler K. Mundinger T. A. Boehm H. Helms H. J. (2013). Adhesion maturation of neutrophils on nanoscopically presented platelet glycoprotein Ibα. ACS Nano 7 (11), 9984–9996. 10.1021/nn403923h 24093566 PMC4122703

[B20] Lee C. Y. Lou J. Wen K. K. McKane M. Eskin S. G. Rubenstein P. A. (2016). Regulation of actin catch-slip bonds with a RhoA-formin module. Sci. Rep. 6, 35058–42322. 10.1038/srep35058 27731359 PMC5059732

[B21] Li N. Yang H. Wang M. Lü S. Zhang Y. Long M. (2018). Ligand-specific binding forces of LFA-1 and Mac-1 in neutrophil adhesion and crawling. Mol. Biol. Cell 29 (4), 408–418. 10.1091/mbc.E16-12-0827 29282280 PMC6014170

[B22] Li Q. Wayman A. Lin J. Fang Y. Zhu C. Wu J. (2016). Flow-enhanced stability of rolling adhesion through E-selectin. Biophys. J. 111 (4), 686–699. 10.1016/j.bpj.2016.07.014 27558713 PMC5002084

[B23] Li R. Emsley J. (2013). The organizing principle of the platelet glycoprotein Ib-IX-V complex. J. Thromb. Haemost. 11 (4), 605–614. 10.1111/jth.12144 23336709 PMC3696474

[B24] Lining J. Yunfeng C. Fangyuan Z. Hang L. Cruz M. A. Cheng Z (2015). Von Willebrand factor-A1 domain binds platelet glycoprotein Ibα in multiple states with distinctive force-dependent dissociation kinetics. Thromb. Res. 136 (3), 606–612. 10.1016/j.thromres.2015.06.019 26213126 PMC4553094

[B25] MacKerell A. D. Bashford D. Bellott M. Dunbrack R. L. Evanseck J. D. Field M. J. (1998). All-atom empirical potential for molecular modeling and dynamics studies of proteins. J. Phys. Chem. B. 102 (18), 3586–3616. 10.1021/jp973084f 24889800

[B26] Marshall B. T. Long M. Piper J. W. Yago T. McEver R. P. Zhu C. (2003). Direct observation of catch bonds involving cell-adhesion molecules. Nature 423 (6936), 190–193. 10.1038/nature01605 Epub 2003/05/09. 12736689

[B27] McDonald B. Pittman K. Menezes B. G. Hirota S. A. Slaba I. Waterhouse C. C. M. (2010). Intravascular danger signals guide neutrophils to sites of sterile inflammation. Science 330 (6002), 362–366. 10.1126/science.1195491 20947763

[B28] McEver R. P. Cummings R. D. (1997). Perspectives series: cell adhesion in vascular biology. Role of PSGL-1 binding to selectins in leukocyte recruitment. J. Clin. Invest. 100 (11 Suppl. l), 485–491. 10.1172/JCI119556 9239393 PMC508213

[B29] Morgan J. Saleem M. Ng R. Armstrong C. Wong S. S. Caulton S. G. (2019). Structural basis of the leukocyte integrin Mac-1 I-domain interactions with the platelet glycoprotein Ib. Blood Adv. 3 (9), 1450–1459. 10.1182/bloodadvances.2018027011 31053572 PMC6517656

[B30] Phillips J. C. Braun R. Wang W. Gumbart J. Tajkhorshid E. (2005). Scalable molecular dynamics with NAMD. J. Comput. Chem. 26 (16), 1781–1802. 10.1002/jcc.20289 16222654 PMC2486339

[B31] Phillipson M. Heit B. Colarusso P. Liu L. Ballantyne C. M. Kubes P. (2006). Intraluminal crawling of neutrophils to emigration sites: a molecularly distinct process from adhesion in the recruitment cascade. J. Exp. Med. 203 (12), 2569–2575. 10.1084/jem.20060925 17116736 PMC2118150

[B32] Rahman S. M. Hlady V. (2019). Downstream platelet adhesion and activation under highly elevated upstream shear forces. Acta Biomater. 91, 135–143. 10.1016/j.actbio.2019.04.028 31004847 PMC6525641

[B33] Sadler J. E. Shelton-Inloes B. B. Sorace J. M. Harlan J. M. Titani K. Davie E. W. (1985). Cloning and characterization of two cDNAs coding for human von Willebrand factor. Proc. Natl. Acad. Sci. U.S.A. 82 (19), 6394–6398. 10.1073/pnas.82.19.6394 2864688 PMC390722

[B34] Schrottmaier W. C. Kral J. B. Badrnya S. Assinger A. (2015). Aspirin and P2Y12 Inhibitors in platelet-mediated activation of neutrophils and monocytes. Thromb. Haemost. 114 (3), 478–489. 10.1160/TH14-11-0943 25904241

[B35] Schwantes C. R. McGibbon R. T. Pande V. S. (2014). Perspective: markov models for long-timescale biomolecular dynamics. J. Chem. Phys. 141 (9), 090901. 10.1063/1.4895044 25194354 PMC4156582

[B36] Silva J. C. Rodrigues N. C. Thompson-Souza G. A. Muniz V. S. Neves J. S. Figueiredo R. T. (2020). Mac-1 triggers neutrophil DNA extracellular trap formation to *Aspergillus fumigatus* independently of PAD4 histone citrullination. J. Leukoc. Biol. 107 (1), 69–83. 10.1002/JLB.4A0119-009RR 31478251

[B37] Simon D. I. Chen Z. Xu H. Li C. Q. Dong J. F. Mcintire L. V. (2000). Platelet glycoprotein Ibalpha is a counterreceptor for the leukocyte integrin Mac-1 (CD11b/CD18). J. Exp. Med. 192 (2), 193–204. 10.1084/jem.192.2.193 10899906 PMC2193258

[B38] Simon D. I. (2012). Inflammation and vascular injury: basic discovery to drug development. Circ. J. 76 (8), 1811–1818. 10.1253/circj.cj-12-0801 22785436 PMC4090145

[B39] Smith G. R. Fitzjohn P. W. Page C. S. Bates P. A. (2005). Incorporation of flexibility into rigid-body docking: applications in rounds 3-5 of CAPRI. Proteins 60 (2), 263. 10.1002/prot.20568 15981258

[B40] Sumagin R. Prizant H. Lomakina E. Waugh R. E. Sarelius I. H. (2010). LFA-1 and Mac-1 define characteristically different intralumenal crawling and emigration patterns for monocytes and neutrophils *in situ* . J. Immunol. 185 (11), 7057–7066. 10.4049/jimmunol.1001638 21037096 PMC3004223

[B41] Torchala M. Moal I. H. Chaleil R. A. G. Fernandez-Recio J., and Bates P. A. (2013). SwarmDock: a server for flexible protein-protein docking. Bioinformatics 29 (6), 807–809. 10.1093/bioinformatics/btt038 23343604

[B42] Von Hundelshausen P. Weber C. (2007). Platelets as immune cells: bridging inflammation and cardiovascular disease. Circ. Res. 100 (1), 27–40. 10.1161/01.RES.0000252802.25497.b7 17204662

[B43] Wang Y. Sakuma M. Chen Z. Ustinov V. Shi C. Croce K. (2005). Leukocyte engagement of platelet glycoprotein Ibalpha via the integrin Mac-1 is critical for the biological response to vascular injury. Circulation 112 (19), 2993–3000. 10.1161/CIRCULATIONAHA.105.571315 16260637

[B44] Whitlock B. B. Gardai S. Fadok V. Bratton V. Henson D. Henson P. M. (2000). Differential roles for alpha(M)beta(2) integrin clustering or activation in the control of apoptosis via regulation of akt and ERK survival mechanisms. J. Cell Biol. 151 (6), 1305–1320. 10.1083/jcb.151.6.1305 11121444 PMC2190581

[B45] Wu T. Lin J. Cruz M. A. Dong J. F. Zhu C. (2010). Force-induced cleavage of single VWFA_1_A_2_A_3_ tridomains by ADAMTS-13. Blood 115 (2), 370–378. 10.1182/blood-2009-03-210369 19897584 PMC2808159

[B46] Yago T. Lou J. Wu T. Yang J. Miner J. J. Coburn L. (2008). Platelet glycoprotein Ibalpha forms catch bonds with human WT vWF but not with type 2B von Willebrand disease vWF. J. Clin. Invest. 118 (9), 3195–3207. 10.1172/JCI35754 18725999 PMC2518822

[B47] Yago T. Wu J. Wey C. D. Klopocki A. G. Zhu C. McEver R. P. (2004). Catch bonds govern adhesion through L-selectin at threshold shear. J. Cell Biol. 166 (6), 913–923. 10.1083/jcb.200403144 15364963 PMC2172126

[B48] Zeng B. Gan Q. Kafafi Z. H. Bartoli F. J. (2013). Polymeric photovoltaics with various metallic plasmonic nanostructures. J. Appl. Phys. 113 (6), 659. 10.1063/1.4790504

[B49] Zhang Y. Lin Z. Fang Y. Wu J. (2020). Prediction of catch-slip bond transition of kindlin2/β3 integrin via steered molecular dynamics simulation. J. Chem. Inf. Model. 60 (10), 5132–5141. 10.1021/acs.jcim.0c00837 32877187

