# Supplementary figures and images for: MD Simulations on a Well-Built Docking Model Reveal Fine Mechanical Stability and Force-Dependent Dissociation of : Mac-1/GPIbα Complex
Source: Front Mol Biosci. 2021 Apr 22;8:638396. doi: 10.3389/fmolb.2021.638396 (PMC8100526; doi:10.3389/fmolb.2021.638396)

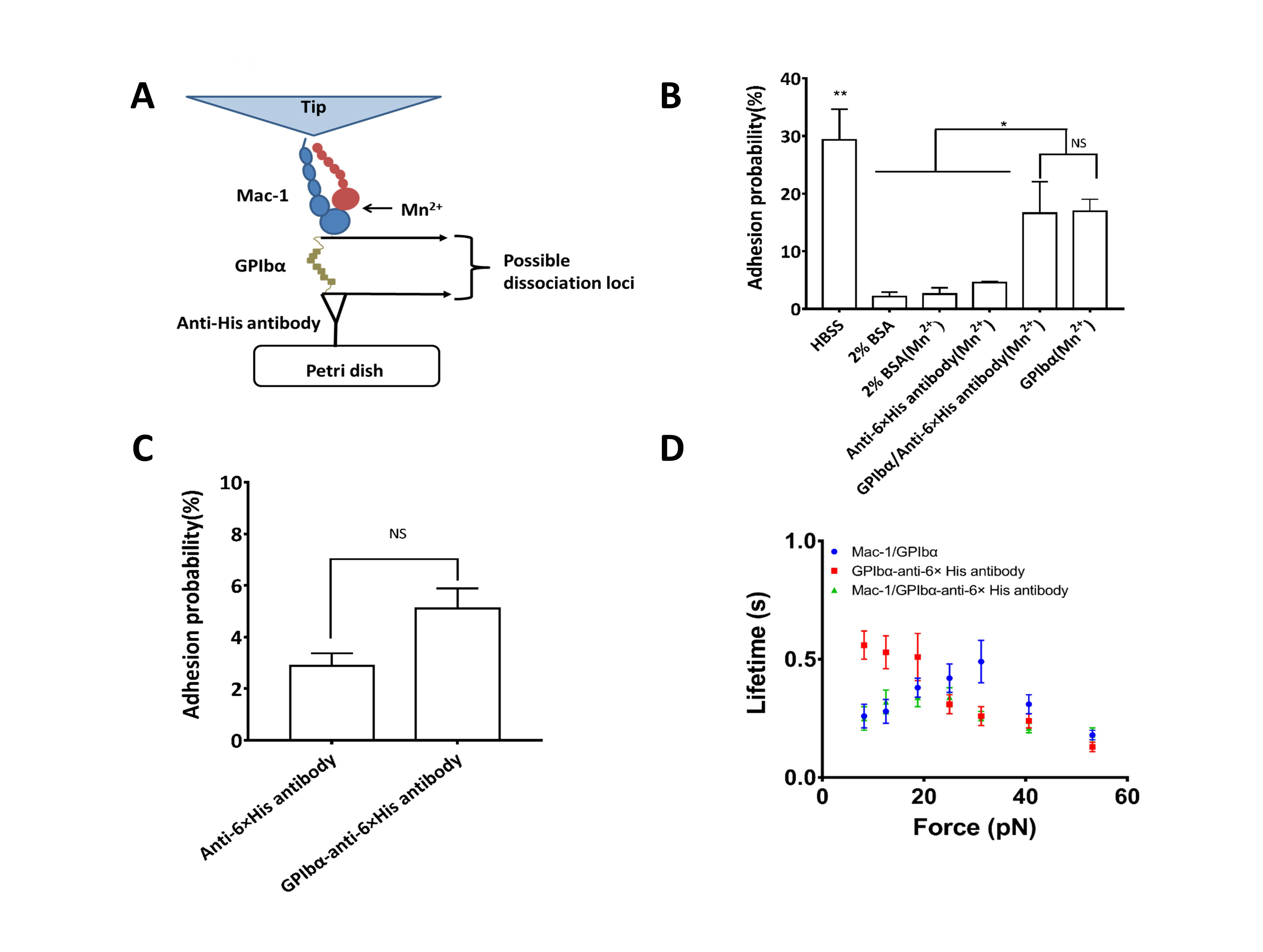

Supplement: Supplementary file 3 [file image3.tif]

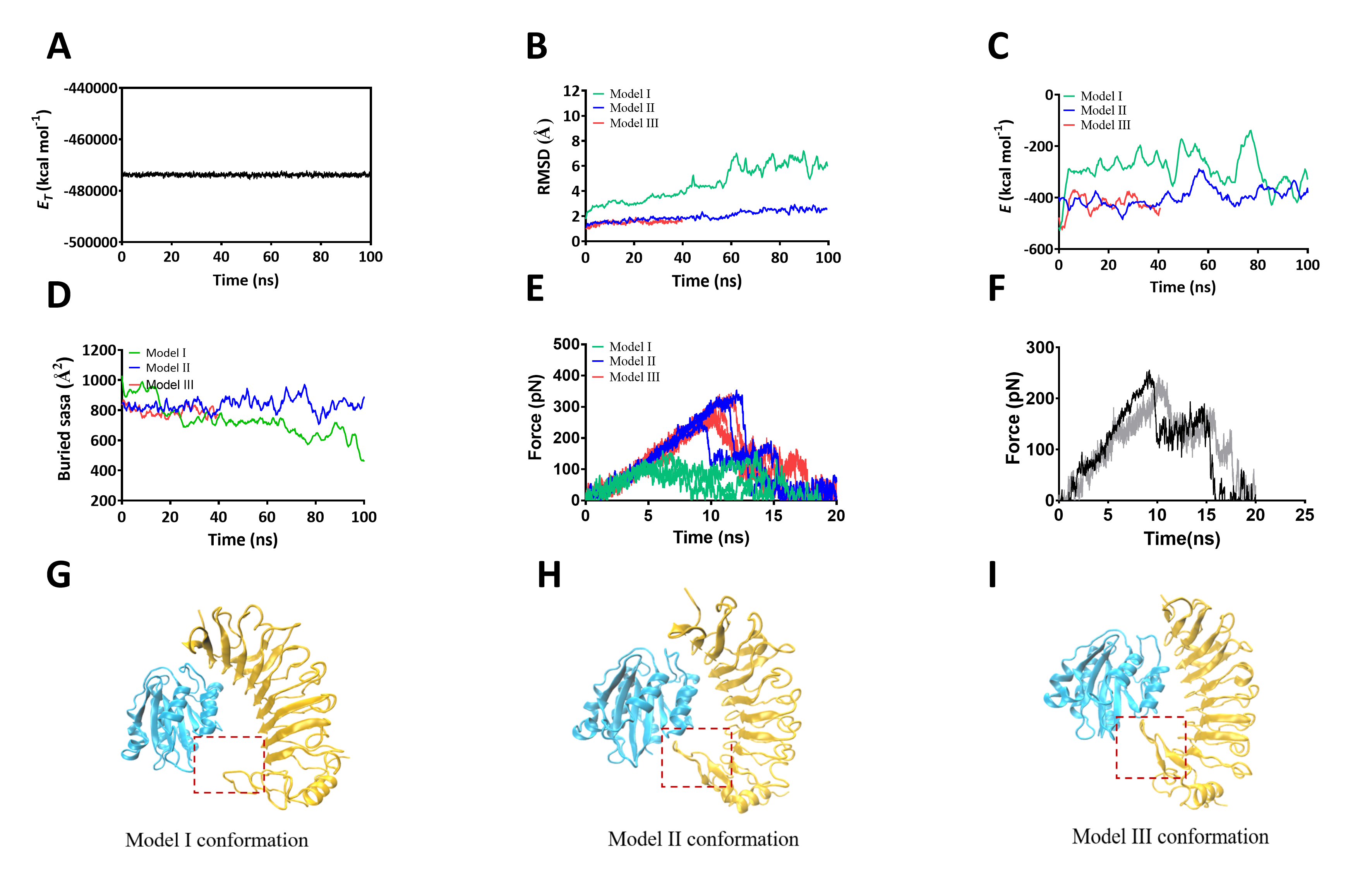

Supplement: Supplementary file 4 [file image2.tif]

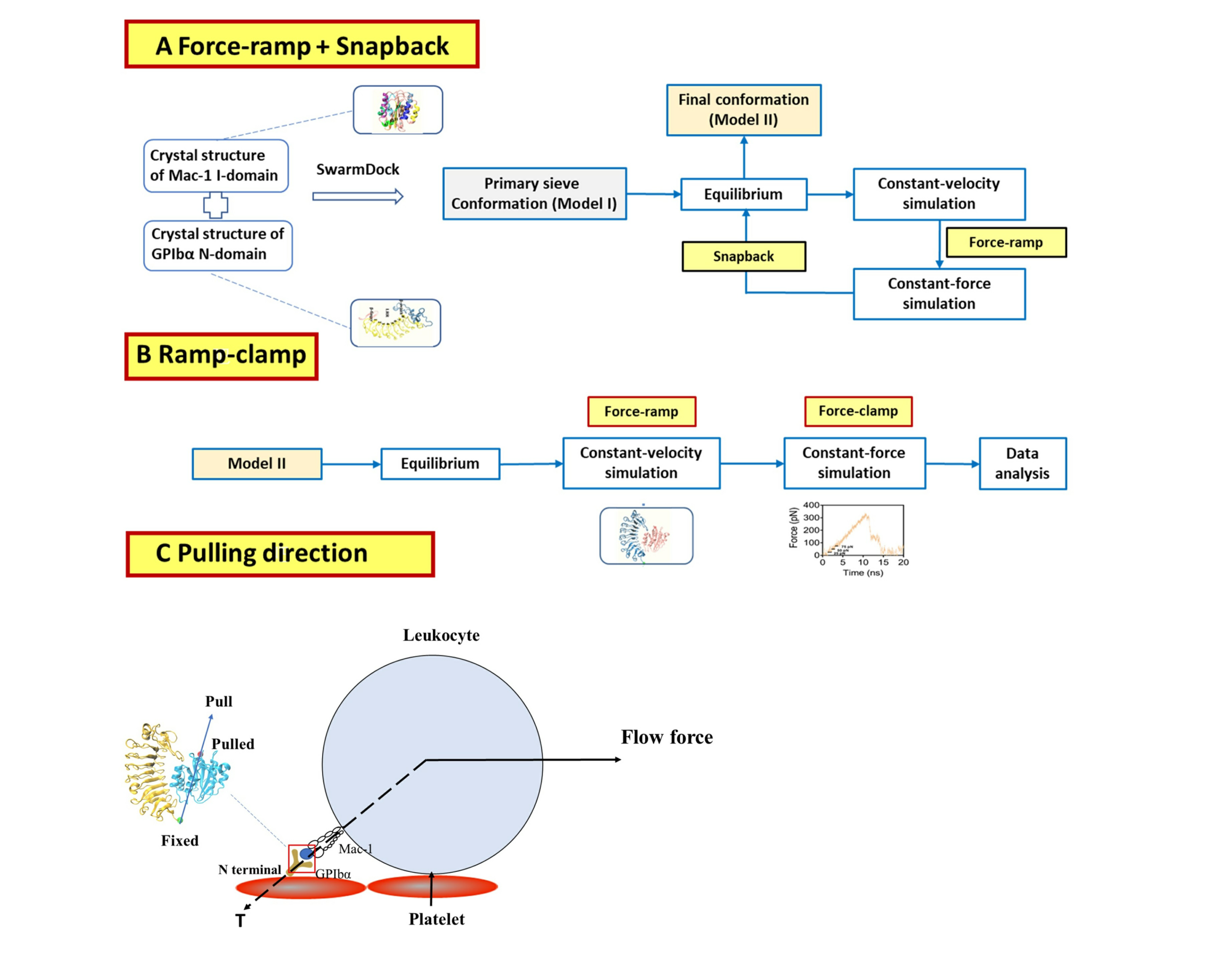

Supplement: Supplementary file 5 [file image1.tif]
