# Supplementary material for: MD Simulations on a Well-Built Docking Model Reveal Fine Mechanical Stability and Force-Dependent Dissociation of : Mac-1/GPIbα Complex
Source: Front Mol Biosci. 2021 Apr 22;8:638396. doi: 10.3389/fmolb.2021.638396 (PMC8100526; doi:10.3389/fmolb.2021.638396)
Supplement: Supplementary file 6 [file datasheet1.pdf]

Supplementary Material

1 Supplementary Figures and Tables

1.1 Supplementary Figures

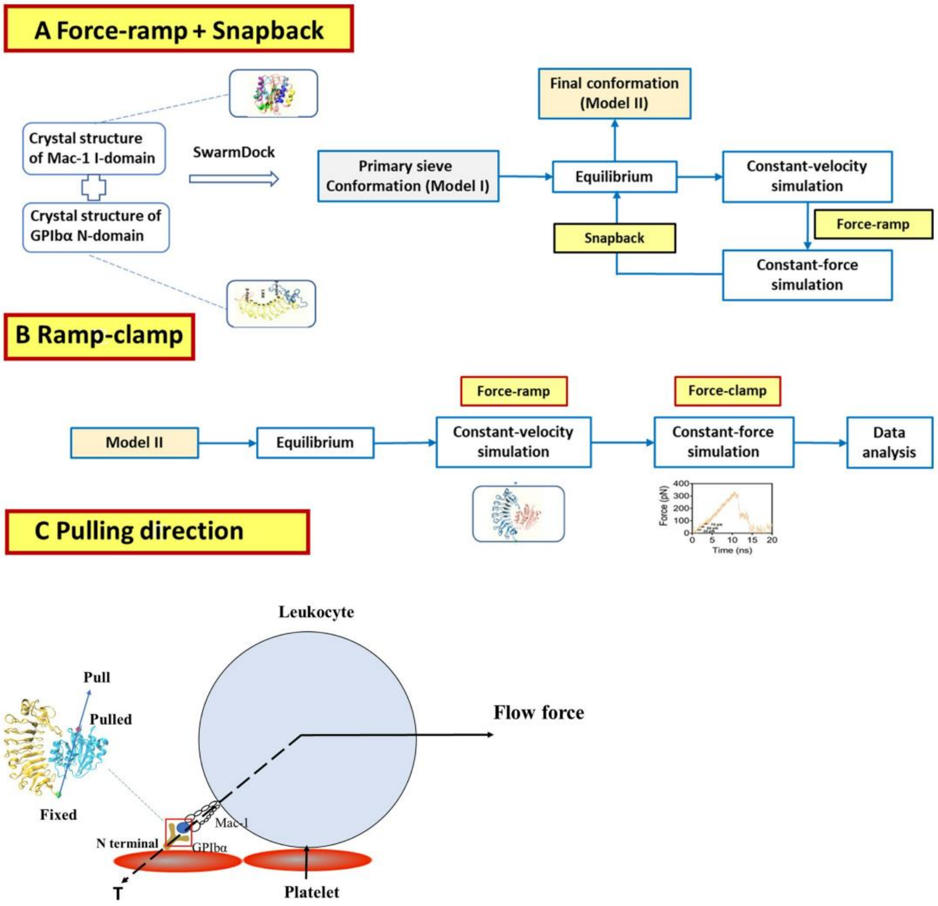

Figure S1. Ensemble workflow of computational procedures. (A-B) Crystal structure of Mac-1 I-domain and GPIbα N were downloaded from the PDB database and were used to dock for the primary sieve conformation (Model I). We first tested the stability and dissociation process of Model I performed by equilibrium and force ramp. We next executed a method named "force-ramp + snapback" to obtain Model II and compared parameters such as interaction energy, RMSD. We finally think that Model II was a more rational conformation. Therefore, the Model II was used to the following force clamp studies. In the simulation process, the structure was solvated with TIP3P water molecules in a rectangular box. The pulling rate was 3 Å/ns during the constant velocity simulations (force-ramp) after 100 ns equilibrium. The Green and red spheres shown are the fixed and steered atoms, respectively. Once the tensile force arrived at a given value (25, 50, and 75 pN), the SMD simulation was transformed from the force-ramp run (constant velocity) mode to a force-clamp (constant force) one. During the constant force simulations, three different initial conformations under forces(25,50,75pN) which were selected randomly for 40 ns simulation. The conformation of 0 pN is equilibrium

conformation. Finally, the results of constant force showed that our method was feasible. **(C)** Schematic diagram of mechanical physiological environment and stretching direction.

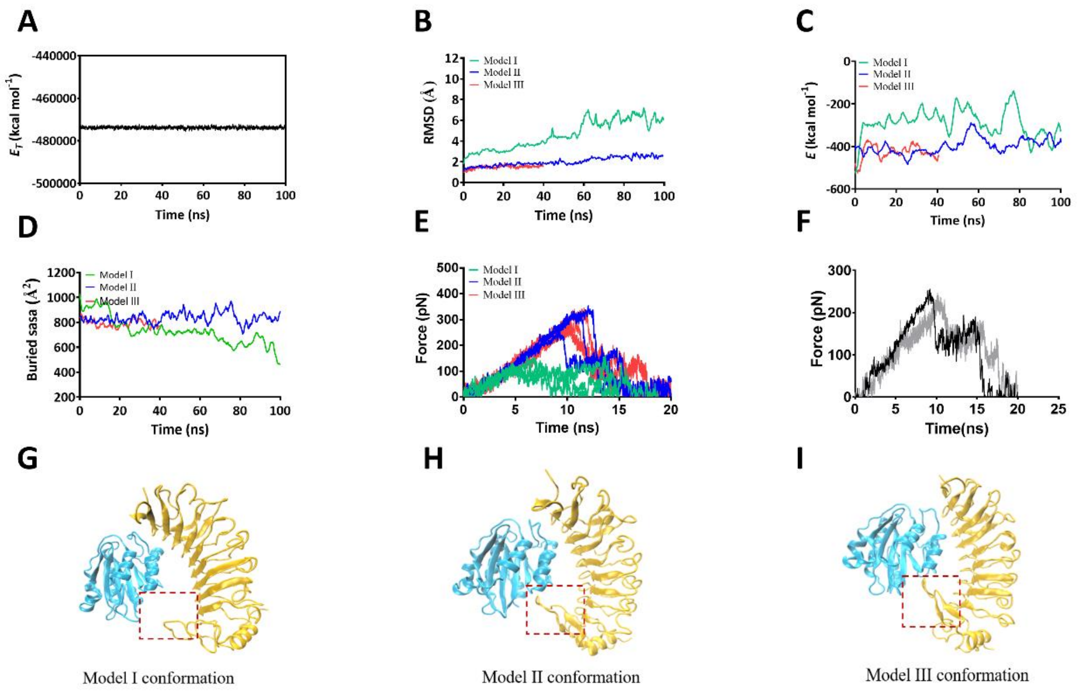

Figure S2. The time courses of three complexes (Model I, Model II, Model III) in the equilibrium and force-ramp and the conformational comparison. (A-D) The time courses of E_T , RMSD, the interaction energies (E), and the buried SASA of Model I , Model II and Model III. **(E)** The time curves of loading force on the complex for three runs. The rupture forces of Model I was about 100-150 pN observed in SMD simulations thrice with a pulling velocity of 3 Å/ns, while the rupture forces of Model II and Model III were about 250-300 pN. The pull direction was fixed the N terminal of GPIbα， pulled the C terminal of Mac-1. **(F)** The time curves of loading force on the complex for Model II. The pull direction was fixed the N terminal of GPIbα, pulled the C terminal of Mac-1 was shown as black. The time curves of loading force which the pull direction was fixed the N terminal of Mac-1, pulled the N terminal of GPIbα was shown as gray. **(G-I)** The conformation comparison of Model I, II and III after the equilibrium simulation. Each point of the force curve was the average force of the preceding 1000 times steps.

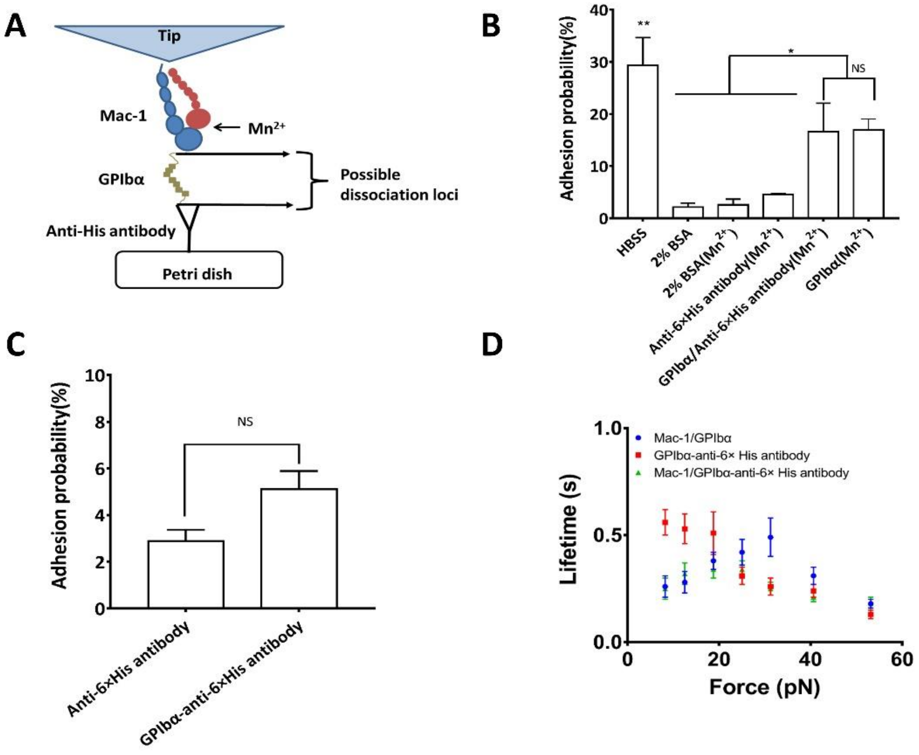

Figure S3. Binding specificity. (A) Possible dissociation loci for Mac-1 and GPIbα-anti-6×His-tag antibody. **(B)** Frequencies of adhesion between Mac-1 adsorbed on cantilever tips and HBSS, HBSS containing 1 % BSA, HBSS containing 2 % BSA, HBSS containing 2 % BSA and 1 mM Mn^{2+}, anti-6×His-tag antibody or GPIbα-anti-6×His-tag antibody precoated on Petri dishes in HBSS containing 2% BSA and 1 mM Mn^{2+}. **(C)** Frequencies of adhesion between HBSS containing 2 % BSA coated on cantilever tips and anti-6×His-tag antibody or GPIbα-anti-6×His-tag antibody precoated on Petri dishes in HBSS containing 2 % BSA and 1 mM Mn^{2+}. Each condition was measured in 100 tests for a single spot and showed as mean ± SEM of 3-4 spots in 3 different experiments. **(D)** Plots of lifetime versus force of Mac-1/GPIbα, GPIbα/anti-6×His antibody and Mac-1/ GPIbα prebound with anti-6×His antibody in HBSS solution containing 2 % BSA and 1 mM Mn^{2+}. The data represent the mean ± SEM from 210-323 records. The significant level of difference is shown by P-value, * $P < 0.05$, ** $P < 0.01$.

1.2 Supplementary Tables

Table S1. H bonds survival occupancy of Model I, Model II and Model III of Mac 1 / GPIbα complex during equilibrium.

No	Residue pair		Occupancy		
	Mac 1	GPIbα	Model I	Model II	Model III
1	K244	D18	0.39	0.77	0.74
2	E282	K19	0.62	0.66	0.67
3	S288	D235	0.23	0.52	0.57
4	E243	K19	0.35	0.44	0.43
5	E252	S39	0.31	0.44	0.53
6	Y251	R64	0.17	0.34	0.53
7	E252	R64	0.003	0.1	0.28
8	E261	K237	0	0.54	0.59
9	R216	D63	0	0.31	0.25
10	K278	Q66	0	0.27	0.23
11	D259	K231	0	0.23	0.15
12	K278	R64	0	0.16	0.12
13	H294	K231	0	0.16	0.2
14	K289	K231	0	0.16	0.18
15	E252	K37	0	0.08	0.03
16	I265	D235	0	0.06	0.04
17	K278	E40	0.67	0.32	0.26

The residual interaction indices in Model I, Model II, and Model III complexes of residual interaction survival ratios measured from equilibrium simulation.

Table S2. The residue pairs on binding site of Mac 1/GPIbα complex under tensile forces.

No.	Residue pair		Force (pN)			
	Mac-1	GPIbα	0	25	50	75
1	E252	K37	0.08	0.19±0.11	0.43±0.15	0.03±0.009
2	E252	S39	0.44	0.67±0.12	0.83±0.03	0.39±0.18
3	E252	R64	0.1	0.74±0.03	0.48±0.23	0.49±0.20
4	E261	K237	0.54	0.58±0.01	0.57±0.03	0.39±0.19
5	I265	D235	0.06	0.19±0.03	0.12±0.03	0.08±0.02
6	K278	E40	0.32	0.62±0.04	0.55±0.08	0.56±0.007
7	S288	D235	0.52	0.79±0.08	0.77±0.04	0.62±0.05
8	R216	D63	0.31	0.04±0.04	0.18±0.04	0.01±0.01
9	K244	D18	0.77	0.61±0.14	0.66±0.03	0.58±0.14
10	Y251	R64	0.34	0.14±0.03	0.28±0.08	0.16±0.09

11	D259	K231	0.23	0.48±0.03	0.25±0.12	0.33±0.16
12	K289	K231	0.16	0.05±0.06	0.18±0.01	0.14±0.01
13	E243	K19	0.44	0.37±0.08	0.24±0.14	0.19±0.08
14	K278	R64	0.16	0.0138±0.012	0.001±0.001	0.002±0.002
15	K278	Q66	0.27	0.04±0.06	0.03±0.02	0.06±0.04
16	E282	K19	0.66	0.64±0.06	0.47±0.1	0.48±0.13
17	H294	K231	0.16	0.02±0.03	0.03±0.01	0.02±0.005

The H-bond occupancies in ranking 1–7 showed catch slip bond over simulation time of 40 ns. Ranking 8-12 displayed three phase changes with force. Ranking 12-17 H-bonds occupancy decreased with forces.

1.3 Supplementary Movies

Supplementary Movies 1 to 2. To examine pull-induced the dissociation of Model I and Model II, we performed the force-ramp SMD simulation over 15 ns thrice with time step of 2 fs and a pulling velocity of 3 Å/ns. The N-terminal Cα atom of GPIbα (residue 1) was fixed, and the C-terminal Cα atom of Mac-1 (residue 317) was steered along pulling direction perpendicular to the binding surface of the complex. The virtual spring, connecting the dummy atom and the steered atom, had a spring constant of 13.48 pN/nm. There Movie was shown a typical dissociation process.

Supplementary Movies 3 to 6. To further examine the regulation of tensile force on exposing of the phosphorylation sites of Mac-1 bound with GPIbα in the force-clamp mode, the so called "ramp-clamp" SMD simulations were performed thrice for tensile forces of 0, 25, 50, and 75 pN. Once tensile force *f* arrived at a given value, such as 0, 25, 50, 75 pN, the SMD simulation was transformed from the force-ramp mode to a force-clamp one, at which the complex was stretched with the given constant tensile force for the followed 40 ns. Each events of hydrogen bonding under stretching were recorded to examine the involved residues and their functions. However, the stretching of 0 pN is derived from equilibrium simulation of Model II; 0, 25, 50, 75 pN correspond to Movie S3, Movie S4, Movie S5, and Movie S6, respectively.
